# Anorexigenic neuropeptides as anti-obesity and neuroprotective agents: exploring the neuroprotective effects of anorexigenic neuropeptides

**DOI:** 10.1042/BSR20231385

**Published:** 2024-04-24

**Authors:** Veronika Strnadová, Andrea Pačesová, Vilém Charvát, Zuzana Šmotková, Blanka Železná, Jaroslav Kuneš, Lenka Maletínská

**Affiliations:** 1 Department of Biochemistry and Molecular Biology, Institute of Organic Chemistry and Biochemistry, Czech Academy of Sciences, Prague, Czech Republic; 2 Department of Biochemistry and Molecular Biology, Institute of Physiology, Czech Academy of Sciences, Prague, Czech Republic

**Keywords:** Alzheimer´s-like pathology, anorexigenic neuropeptides, antiobesity treatment, neuroprotection

## Abstract

Since 1975, the incidence of obesity has increased to epidemic proportions, and the number of patients with obesity has quadrupled. Obesity is a major risk factor for developing other serious diseases, such as type 2 diabetes mellitus, hypertension, and cardiovascular diseases. Recent epidemiologic studies have defined obesity as a risk factor for the development of neurodegenerative diseases, such as Alzheimer’s disease (AD) and other types of dementia. Despite all these serious comorbidities associated with obesity, there is still a lack of effective antiobesity treatment. Promising candidates for the treatment of obesity are anorexigenic neuropeptides, which are peptides produced by neurons in brain areas implicated in food intake regulation, such as the hypothalamus or the brainstem. These peptides efficiently reduce food intake and body weight. Moreover, because of the proven interconnection between obesity and the risk of developing AD, the potential neuroprotective effects of these two agents in animal models of neurodegeneration have been examined. The objective of this review was to explore anorexigenic neuropeptides produced and acting within the brain, emphasizing their potential not only for the treatment of obesity but also for the treatment of neurodegenerative disorders.

## Introduction

**Figure 1:**
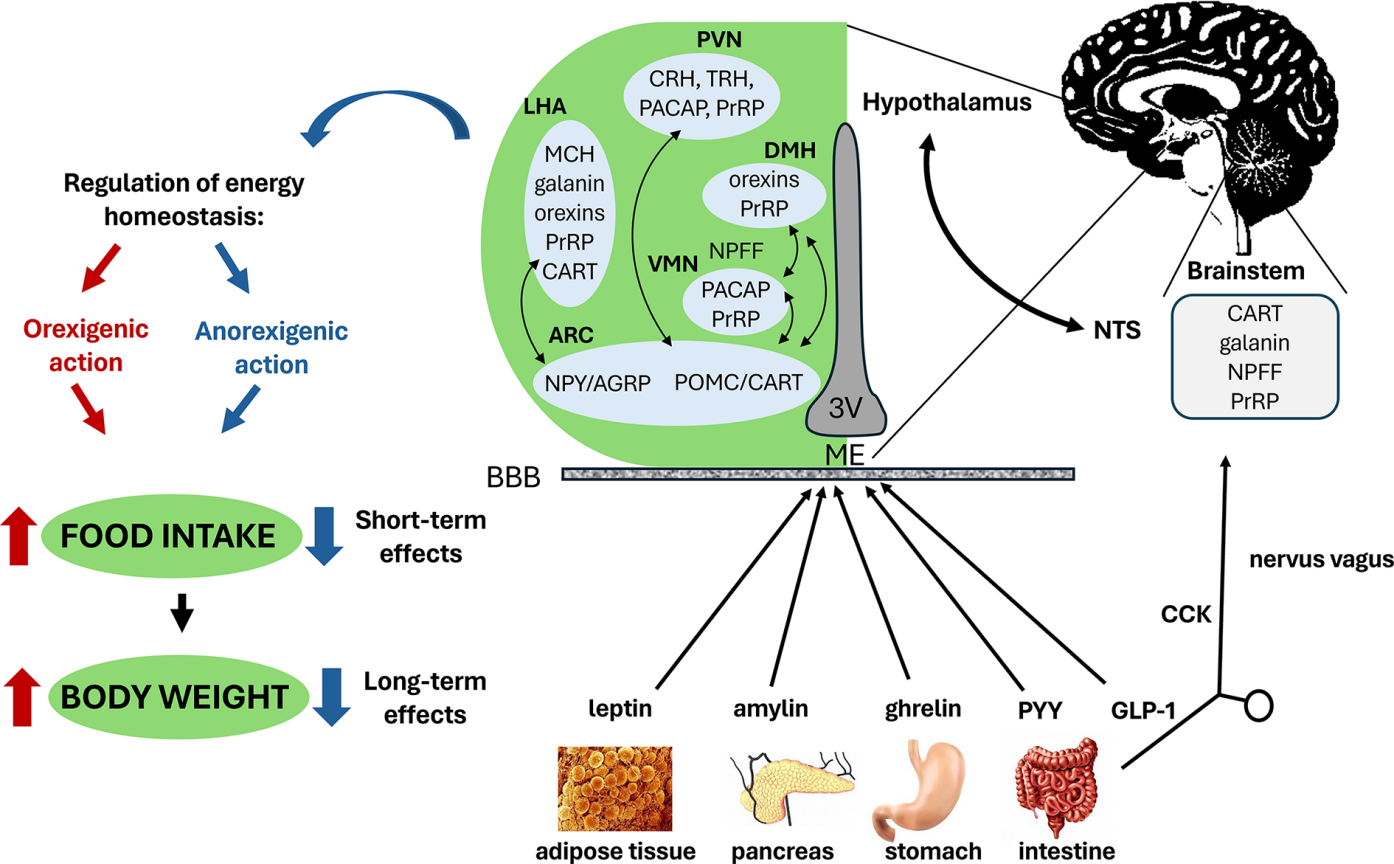
Scheme of peptides involved in food intake regulation Food intake regulating peptides are produced in the periphery, as well as in the brain. The main regulators of energy homeostasis are anorexigenic (food intake lowering) leptin, produced by white adipose tissue, and orexigenic (food intake stimulating) ghrelin, produced by the stomach. Many other anorexigenic hormones are produced in the gastrointestinal tract, for example, amylin, cholecystokinin (CCK), glucagon-like peptide 1 (GLP-1), or peptide YY (PYY). To induce biological effects, these peptides must penetrate to the brain; either through nervus vagus to the nucleus of the solitary tract (NTS) or through the blood–brain barrier (BBB) to the hypothalamus that is the main center of food intake regulation. In the arcuate nucleus (ARC), located at the base of the third ventricle (3V) adjacent to the media eminence (ME), two distinct populations of neurons regulate food intake with antagonistic effects: anorexigenic pro-opiomelanocortin (POMC) and cocaine- and amphetamine-regulated transcript (CART) peptide, alongside orexigenic neuropeptide Y (NPY) and agouti-related peptide (AgRP). These neuronal populations project to other hypothalamic nuclei, such as ventromedial nucleus (VMN), dorsomedial nucleus (DMN), lateral hypothalamic area (LHA), and paraventricular nucleus (PVN), where are expressed other anorexigenic (corticotropin-releasing hormone [CRH], neuropeptide FF [NPFF], pituitary adenylate cyclase-activating peptide [PACAP], prolactin-releasing peptide [PrRP], and thyrotropin-releasing hormone [TRH]) or orexigenic neuropeptides (galanin, melanin-concentrating hormone [MCH], orexins).

The regulation of food intake and energy homeostasis is a very complex process in which both central and peripheral mechanisms are involved [1]. The central nervous system (CNS), mainly the hypothalamus, is a key regulator of energy homeostasis and is responsible for coordinating physiological processes related to hunger and satiety to maintain energy balance through long-term and short-term signals [2]. These signals are integrated and further processed in the arcuate nucleus (ARC) which is a nucleus at the base of the third ventricle adjacent to the media eminence (ME), one of the circumventricular organs with fenestrated capillaries that allows the transport of different molecules and hormones from the periphery to the brain [3,4]. The ARC contains two distinct populations of neurons with antagonistic properties. The first population produces food intake-stimulating orexigenic neuropeptides, such as neuropeptide Y (NPY) and agouti-related peptide (AgRP), while the second population produces food intake suppressing anorexigenic pro-opiomelanocortin (POMC) and cocaine- and amphetamine-regulated transcript (CART) peptide [5]. One of the peripheral hormones that influence the expression of these neuropeptides in the ARC is leptin, the main regulator of energy balance produced in white adipose tissue (WAT) [6]. Its level in the blood plasma is proportional to the total amount of WAT [7]. Leptin decreases food intake by inhibiting orexigenic NPY/AgRP neurons and simultaneously stimulating anorexigenic POMC/CART neurons [8]. Moreover, leptin decreases the accumulation of fat in the body [9]. This effect of leptin is an example of cooperation among peptides from the periphery and neuropeptides (Figure 1). Another important peripheral hormone that regulates ARC neuropeptides is ghrelin, the only known peripheral orexigenic compound produced in the stomach [10]. Other anorexigenic peptide hormones important for the regulation of food intake, such as amylin [11], cholecystokinin (CCK) [12], glucagon-like peptide 1 (GLP-1) [13], or peptide YY (PYY) [14], are secreted in the periphery (reviewed [15–17]). However, the objective of this review is to focus on neuropeptides, defined as peptides produced within the nervous system that are released by various populations of neurons and function within the brain.

Neuropeptides produced from the ARC influence other hypothalamic areas, such as the paraventricular nucleus (PVN), ventromedial nucleus (VMN), dorsomedial hypothalamic nucleus (DMH), and lateral hypothalamic area (LHA) thereby affecting the release of other neuropeptides, both orexigenic (galanin, melanin-concentrating hormone [MCH], and orexins) [18–20] and anorexigenic (corticotropin-releasing hormone [CRH], pituitary adenylate cyclase-activating peptide [PACAP], prolactin-releasing peptide [PrRP], and thyrotropin-releasing hormone [TRH]) [21–25]. From these areas the impulses are projected to the thalamus [26] and are integrated with signals from the brainstem, mainly from the nucleus of the solitary tract (NTS) [27]. When the balance between energy input and output works, physiological equilibrium is maintained. There is currently no effective system for monitoring the caloric intake of individual organs and tissues. Consequently, monitoring fat store bulkiness could be an appropriate approach; if these stores remain unchanged, the energy balance is satisfactory [28]. However, if there is excess energy stored in fat tissue, obesity and its associated comorbidities, such as type 2 diabetes mellitus (T2DM), hypertension, cardiovascular diseases, and metabolic syndrome [8,29,30], develop. Recently, obesity has also been associated with the development of neurodegenerative diseases [31] representing a significant and escalating global challenge. Typically, neurodegenerative diseases exhibit delayed onset, and progressive clinical symptoms and are characterized by neuronal loss [32]. This neuronal loss is known as brain atrophy and leads to memory impairment and dementia [33,34]. Among all neurodegenerative diseases, the most common type is Alzheimer’s disease (AD), which accounts for 60–80% of all cases of dementia according to the World Health Organization [35]. AD is characterized by presence of senile plaques formed by amyloid-β (Aβ), Tau protein hyperphosphorylation, increased neuroinflammation, and decreased synaptogenesis and neurogenesis in the brain [36]. In dementias, including AD, neurodegeneration is associated with aging, leptin and insulin resistance [37,38], inflammation mediated by cytokines [39], oxidative and cellular stress [40], cell death, vascular destruction [41], or dysregulation of the energy balance and metabolism of neuropeptides involved in food intake regulation [42]. These features are also observed in T2DM [43], metabolic syndrome [44], and nonalcoholic fatty liver disease [45]. Therefore, these diseases could be managed through similar, if not identical, therapeutic strategies. Thus, drugs initially developed for obesity treatment might also be useful for the treatment of AD [46], supporting the critical role of neuropeptides in the regulation of neuronal activity [47,48].

While central administration of anorexigenic neuropeptides efficiently decreases food intake and body weight, peripheral application triggers no response due to the inability of neuropeptides to penetrate the brain, where their receptors are expressed. To achieve central biological effects after peripheral administration, such as the abovementioned decrease in food intake, it is necessary to modify neuropeptides to enhance their stability and bioavailability. One example of the improved properties of modified peptides is long-lasting receptor agonists of GLP-1, such as liraglutide, exenatide or lixisenatide, which are on the market as antidiabetic and antiobesity drugs, and currently, their neuroprotective properties are also being investigated in clinical trials [49,50]. In preclinical studies, treatment with liraglutide reduced Aβ plaques, Tau hyperphosphorylation, and neuroinflammation. Additionally, it increases synaptic plasticity, neurogenesis and enhanced memory [51–54]. However, manipulating the central neuropeptide system poses a real therapeutic challenge, as peripherally injected neuropeptides need to access the brain without causing any side effects.

The objective of this review is to summarize the current knowledge on anorexigenic neuropeptides that are produced and acting within the brain, and their modified analogs capable to act centrally after peripheral administration in animal preclinical models with an emphasis on their possible use for treating obesity and neurodegenerative disorders.

## Orexigenic neuropeptides

Orexigenic neuropeptides, such as NPY, AgRP, MCH, orexins, and galanin, stimulate food intake. Despite their opposite effects on the food intake regulation, orexigenic peptides, like anorexigenic peptides, have shown neuroprotective effects on preclinical models of neurodegenerative diseases. However, they are not the focus of this review.

Decreased NPY levels in different brain regions and plasma have been described in several preclinical models of AD, as well as in patients with AD [55]. NPY can function as an antiapoptotic, anti-inflammatory and neuroprotective agent, as reviewed in [48,56]. Moreover, NPY, implicated in stress response, anxiety, and cognition, also plays an important neuroprotective role in neurodegenerative diseases like Alzheimer’s, Parkinson’s, and Huntington’s disease, where stress is a contributing factor [57]. In the Aβ mouse model of AD, a sole intracerebroventricular (ICV) injection of NPY mitigates depressive-like behavior, spatial memory deficits, and oxidative stress induced by Aβ administration [58]. In addition to its role in regulating energy balance, AgRP, produced by AgRP/NPY neurons, exerts influence on various cellular processes. Notably, the ubiquitin proteasome system, crucial for the targeted degradation of short-lived proteins, is typically downregulated in AD. Lee at al. [59] demonstrated that the recombinant human AgRP protein increases proteasome activity in SH-SY5Y cells. Additionally, in 5xFAD mice, administration of AgRP protein led to an increase in proteasome activity and inhibited the accumulation of ubiquitin-conjugated proteins. This suggests that AgRP has the potential to decrease abnormal protein aggregation, thereby potentially slowing down the clinical progression of various neurodegenerative diseases [59].

The orexigenic neuropeptide MCH is produced in LHA neurons that further project to the hippocampal *cornu ammonis* (CA) 1 area, which is connected to memory formation. Mice with knock-in Swedish mutation in amyloid precursor protein (APP), which leads to the development of the familial form of AD (APP^NL-G-F^ mice), presented a decreased MCH level and subsequent aberrant excitation of hippocampal neurons. Thus, MCH deregulation may be involved in the development of the early stages of AD [60]. In the study by Oh et al. [61], MCH peptide was intranasally administered to scopolamine-induced memory-impaired mice to assess acute effects and to AD mouse models to investigate chronic effects. MCH ameliorated memory impairment in these models and reduced soluble Aβ levels in the cerebral cortex of APP/PS1 transgenic mice. Additionally, MCH enhanced long-term potentiation in the hippocampus of both wild-type and 5xFAD AD mouse models [61]. The administration of MCH peptide into the hippocampus and amygdala enhanced the memory performance of rats [62] and reverse the amnesic effects induced by a nitric oxide synthase inhibitor [63], a known disruptor of hippocampal plasticity. N-methyl-D-aspartate (NMDA) receptors play a pivotal role in the remarkable plasticity exhibited by the hippocampus [64] and are fundamentally implicated in the neural mechanisms that underlie specific forms of learning. In hippocampal slices from rats treated with MCH and subjected to a memory task, an increase in the expression of NMDA receptor subunits crucial for synaptic plasticity was observed [64].

Orexin A and orexin B produced in the LHA are involved in sleep/wake processes, appetite, drug addiction and cognitive processes. Orexins also have neuroprotective and anti-inflammatory properties, as reviewed previously [65,66]. Previous studies have indicated that orexin-A exhibits protective effects in cellular models of Parkinson’s disease. Liu et al. revealed that orexin-A mitigated the loss of dopaminergic neurons and the reduction in tyrosine hydroxylase expression in the substantia nigra in a mouse model of Parkinson’s disease. Orexin-A improved both motor activity and spatial memory in this mouse model and elevated the protein levels of brain-derived neurotrophic factor (BDNF), which promotes neuroprotection and neuroregeneration, in dopaminergic neurons of the substantia nigra [67].

In addition to its orexigenic effect, galanin is involved in a broad range of physiological functions including effects on memory, learning and neurogenesis in the hippocampus [68]. The intranasal coadministration of galanin receptor-2 (GALR2) agonist (M1145) and NPY receptor 1 (NPY1R) agonist improved spatial memory in the Sprague-Dawley rats [68]. Subsequent study revealed a sustained increase in neurogenesis in the dorsal dentate gyrus following ICV administration of GALR2 and NPY1R agonists. Simultaneous delivery of the M1145 and the NPY1R agonist promoted neuroblast proliferation and improvement in object-in-place memory [69]. Additionally, galanin receptor-2/3 agonist (Gal 2-11) induced proliferation of hippocampal precursor cells, thus directly affected hippocampal neurogenesis, impaired in AD, through production of granule cell neurons of the dentate gyrus [70].

## Anorexigenic neuropeptides

Anorexigenic neuropeptides inhibiting food intake include POMC, CART peptide (CARTp), PACAP, PrRP, neuropeptide FF (NPFF), CRH, and THR. The potential of these peptides in the treatment of obesity and neurodegeneration is described in separate chapters. The order was determined according to the expression in individual hypothalamic nuclei.

### Melanocortin system

The melanocortin system includes multiple peptides such as α, β and γ-melanocyte-stimulating hormone (MSH), adrenocorticotrophic hormone (ACTH), and β-lipotropin, which are derived from the precursor POMC [71,72]. The precursor protein POMC consists of three main domains whose different cleavages by proprotein convertases produce different molecules, such as γ-MSH from the N-terminal region, ACTH from the central region, which can be further cleaved to α-MSH, and β-MSH and β-endorphin cleaved from β-lipotropin from the C-terminal domain [73]. The posttranslational processing of POMC occurs in a tissue-specific manner and results in diverse biological functions. The POMC system controls nervous, behavioral, endocrine, and immune functions and has a regulatory and homeostatic roles [71].

These molecules are cleaved from POMC and share the common tetrapeptide core sequence His-Phe-Arg-Trp which interacts with and activates melanocortin receptors (MCRs). MCRs are G protein-coupled receptors (GPCR) that include five receptor variants with multiple physiological functions. MC1R regulates pigmentation in melanocytes, MC2R activates glucocorticoid biosynthesis in the adrenal cortex, MC3R and MC4R influence energy homeostasis in the central nervous system, and MC5R regulates the synthesis and secretion of exocrine gland products [74,75]. ACTH and α-MSH can activate MC1R, MC3R, MC4R, and MC5R. However, MC2R can be activated by ACTH but not any other melanocortin [74,76]. Additionally, MC3R has a very high binding capacity for γ-MSH, while MC4R has a very high binding capacity for α- and β-MSH [77]. Orexigenic AgRP is a natural antagonist of MC3R and MC4R activity [78,79].

POMC is highly expressed in endocrine cells of the pituitary gland and neurons of the hypothalamic ARC [75,80]. Melanocortins are part of the anorexigenic system that decreases appetite and food intake, therefore they play an important role in the regulation of body weight homeostasis and energy balance. The primary effects of POMC-derived peptides on feeding and body weight are mediated by MSH peptides and their effects on MC3R and MC4R [81]. MC3R and MC4R have been investigated as promising targets for anti-obesity drugs [82–84]. Humans deficient in POMC or MC4R are hyperphagic and severely obese [85,86]. Additionally, MC3R mutation variants cause robust obesity in humans [77]. An obese phenotype is also evident in knockout (KO) MC3R and MC4R mice. While MC4R KO mice exhibit hyperphagia and develop T2DM, MC3R KO mice are not hyperphagic and have a normal metabolic response [87].

α-MSH, produced in the ARC and acting at MC4R in the PVN is important for the regulation of food intake and energy balance and is one of the main mediators of the effects of leptin [72]. The ICV administration of melanotan II (MTII) a cyclic melanocortin agonist with the sequence Ac-Nle^4^-c[Asp^5^,D-Phe^7^,Lys^10^]α-MSH- [4–10]-NH_2_ resulted in a significant reduction in food intake and body weight in male Sprague-Dawley rats fed a high-fat diet (HFD) [88]. Interestingly, consumption of a HFD decreases signaling through the melanocortin system. Rats maintained on a HFD were less sensitive to the inhibition of food intake induced by MTII [89]. MTII is a potent agonist of both MC3R and MC4R, and when MTII is administered via the ICV, it inhibits acute food intake in fasted mice [90] but has no effect on food intake in fasted Mc4r^–/–^ mice [91,92]. Another MC4R peptide agonist, the lipidized analog of α-MSH [93], called MC4-NN1-0182, was investigated in rats with diet-induced obesity (DIO) and DIO minipigs. Long-term treatment with MC4-NN1-0182 resulted in a decrease in food intake and body weight [94]. Moreover, DIO rats exhibit reduced levels of leptin, cholesterol, and insulin, with a slight increase in oxygen consumption [94]. α-MSH activates a thermogenic gene program and increases the mitochondrial respiratory rate in adipocytes and inguinal WAT of DIO mice. Without affecting food intake, peripheral administration of α-MSH decreased body weight and inguinal WAT mass [95].

Another melanocortin analog, cyclic peptide setmelanotide (also known as BIM-22493), demonstrated a reduction in acute food intake in fasted mice. Interestingly, the inhibition of refeeding after an overnight fast by BIM-22493 was dependent on functional MC4R and did not require MC3R [96]. Chronic treatment of DIO mice with BIM-22493 resulted in weight loss and improvements in hyperinsulinemia and fatty liver. However, treatment with BIM-22511 did not impact body weight in MC4R KO mice but did reduce body weight in MC3R KO mice. Additionally, chronic treatment with BIM-22511 did not improve hepatosteatosis in MC4R KO mice and did not affect hepatic lipogenic gene expression. MC4R is necessary for melanocortin agonist-induced weight loss and improvements in liver metabolism but is not required for improvements in hyperinsulinemia [96].

α-MSH and its analogs have been proposed to exhibit neuroprotective and anti-inflammatory effects and represent a potential strategy for treating AD [97]. Concentrations of α-MSH in the brain and cerebrospinal fluid of AD patients were reduced, correlating with cognitive dysfunction [98,99]. Activation of POMC-derived neuropeptides and MCRs has previously been shown to rescue the impairment of synaptic plasticity in a mouse model of AD. Treatment with α-MSH preserved the expression of the GABAergic marker GAD67 (glutamic acid decarboxylase 67) promoted the survival of GABAergic GAD67+ inhibitory interneurons in the hippocampus and improved spatial memory in the TgCRND8 mouse model of AD with Swedish and Indiana mutations in APP [100]. In ischemic rats, which display a reduced number of neurons with pathological morphological changes in the CA1 pyramidal cell layer of the hippocampus, treatment with α-MSH leads to an increase in the number of viable hippocampal neurons. Additionally, α-MSH decreases glial activation, as indicated by the reduction in glial fibrillary acidic protein (GFAP), an astrocyte marker that is markedly elevated in ischemic rats. Therefore, the neuroprotective effect of α-MSH could be attributed to the reduction of damage caused by reperfusion. However, further studies will be necessary to determine whether the neuroprotective effect of α-MSH is mediated by its anti-inflammatory actions [101].

The melanocortin analog [Nle^4^, D-Phe^7^]α-MSH (NDP-α-MSH) has been shown to improved learning and memory, as well as increase neurogenesis in Mongolian gerbils [102]. The effect of chronic administration of NDP-α-MSH was investigated in several mouse models of AD – in 3xTg mice, a model of AD containing three human mutations, APP_Swe_, presenilin 1 (PS1)_M146V_, and Tau_P301L_ [103,104]; in Tg2576 mice carrying the APP_SWE_ mutation [105,106]; and in 5XFAD mice with 5 mutations connected to AD-Swedish (K670N/M671L), Florida (I716V), and London (V717I) mutations in APP, and the M146L and L286V mutations in PS1 [104]. NDP-α-MSH improved spatial memory in Morris water maze (MWM) in all mentioned AD mouse models [103–106]. NDP-α-MSH further reduced the level of Aβ deposits in Tg2576 [105] and in 30-week-old 3xTg mice [103]. However, the level of Aβ deposits or astrocytic reactivity were not influenced by NDP-α-MSH in 9- and 14-month-old 3xTg mice or in 5XFAD mice [104]. While NDP-α-MSH did not influence microglial reactivity, as indicated by ionized calcium binding adaptor molecule 1 (Iba1) staining, in 5XFAD mice, it reduced microglial reactivity in the CA3 region in 3xTg mice [104]. Treatment also attenuated Tau hyperphosphorylation at different epitopes in 3xTg and 5XFAD mice [103,104] and reduced the level of p38 mitogen-activated protein kinase (MAPK), which is a kinase that is overactivated in patients with AD [107]. Finally, NDP-α-MSH decreased neuronal loss and increased hippocampal expression of the immediate early response gene Zif268, suggesting increased synaptogenesis [103,105]. Moreover, it increased the number of bromodeoxyuridine immunoreactive cells, a marker of cell proliferation, which are colocalized with the markers of mature neurons NeuN and Zif268 in the hippocampus of Tg2576 mice [106]. The central administration of another MCR agonist [d-Tyr^4^]-melanotan II, reduced Aβ levels, improved inflammation and astrocytic activation in the hippocampus and suppressed microglial activation in APP/PS1 mice. It significantly reduced 6E10-immunostained amyloid plaques and decreased levels of both insoluble and soluble Aβ, with reduced levels of isomers Aβ_x–42_ and Aβ_x–40_. Additionally, the treatment lowered the elevated expression of pro-inflammatory factors interleukin-1β (IL-1β) and interleukin-6 (IL-6), as well as anti-inflammatory cytokine intercellular adhesion molecule 1 (Icam1). Moreover, [d-Tyr^4^]-melanotan II reduced the increased expression and immunoreactivity of GFAP, particularly in the CA1 zone, and decreased microglial density in the hippocampus [108].

Considering these results, which are summarized in Table 1 and in Table 2, α-MSH treatment represents a strategy for treating obesity, improving cognitive function, and exerting neuroprotective effects along with increased neurogenesis.

**Table 1:** Effect on food intake and body weight after chronic administration of neuropeptides or their modified analogs in DIO rodent models

Animal model HFD: starting age and weeks of feeding	Intervention Compound/ dose/ injection/ duration	Effects Increased ↑ Decreased ↓	Reference
	**Melanocortins**		
**Sprague-Dawley rats ♂** HFD: from 6th week, 12 weeks	**MTII** 0.5 nmol/rat, ICV once	Reduced food intake Reduced body weight	[88]
**Long-Evans rats ♂** HFD: from 6th week, 8 weeks	**MTII** 0.1, 0.3, 1.0 nmol/rat, ICV once	Reduced food intake	[89]
**Sprague-Dawley rats ♂** HFD: from 7 to 8th week, 10 weeks **Göttingen minipigs ♀** NS *ad libitum* fed half a year	**MC4-NN1-0182** 0.5 ml/kg, SC 23 days 30 mg/pig at day 0, SC 10 mg/pig every other day 58 days	Reduced food intake Reduced body weight and adipose tissue Leptin, cholesterol, and insulin ↓ Reduced food intake Reduced body weight	[94]
**C57BL/6 mice ♂** HFD: from 10th week, 10 weeks	**α-MSH** 150 μg/kg, IP 14 days	Reduced body weight and ingWAT *Ucp1* mRNA ↑ *Pgc-1α* mRNA ↑ Thermoregulatory-related genes ↑	[95]
**C57BL/J DIO mice ♂** HFD: NS **MC3R KO mice ♀** STD: NS **MC4R KO mice ♀** STD: NS	**BIM-22493** 300 nmol/kg/day, SC osmotic pump 14 days **BIM-22511** 100 nmol/kg/day, SC osmotic pump 14 days **BIM-22511** 100 nmol/kg/day, SC osmotic pump 14 days	Reduced food intake Reduced body weight Leptin, cholesterol, and insulin ↓ Improved liver steatosis Reduced body weight No reduction of body weight Insulin ↓	[96]
	**CART peptide**		
**Long-Evans rats ♂** NS	**CARTp [55–102]** 9 µg/day, ICV 6 days	Reduced food intake Reduced body weight Leptin, insulin, glucose ↓	[141]
**Sprague-Dawley rats ♂** HFD: NS, 3 weeks	**CARTp [55–102]** 500 pmol/each two ICV injections	Reduced food intake Lipid metabolism ↑ NEFA ↑	[142]
	**PrRP**		
**C57BL/6 mice ♂** HFD: from 8th week, 12 weeks	**Palm-PrRP31 or Myr-PrRP20** 5 mg/kg, SC twice a day 14 days	Reduced food intake Reduced body weight Reduced amount of WAT Leptin ↓, Insulin (only palm-PrRP31) ↓ *Fasn* mRNA↓	[177]
**C57BL/6 mice ♂** HFD: from 8th week, 12 weeks	**Palm^11^-PrRP31** 5 mg/kg, SC twice a day 2 weeks	Reduced food intake Reduced body weight Reduced amount of scWAT Insulin, Leptin, TAG, FFA, cholesterol ↓ *Fasn* m RNA in WAT ↓ *Ucp1* mRNA in BAT ↑	[179]
**C57BL/6 mice ♂** HFD: from 8th week, 12 weeks	**Palm^11^-PrRP31** 5 mg/kg, SC twice a day 28 days, or 14 days + 14 days wash-out	Reduced food intake of both groups Reduced body weight of both groups Reduced amount of scWAT Leptin ↓ pAkt Ser473, p-ERK, PI3K in the hypothalamus ↑ *Ucp1* mRNA in BAT ↑	[180]
**Wistar Kyoto rats ♂** HFD: from 8th week, 15 weeks	**Palm^11^-PrRP31** 5 mg/kg, IP once a day 21 days	Reduced body weight Reduced amount of WAT Improved OGTT *Acaca* mRNA, *Fasn* mRNA in scWAT ↓	[181]
**Sprague–Dawley rats ♂** HFD: from 7th to 9th week, 24 weeks	**Palm-PrRP31** 1 or 5 mg/kg, IP once a day 15 days	Reduced food intake Reduced body weight	[182]
**Wistar Kyoto rats ♂** HFD: from 8th week, 52 weeks	**Palm^11^-PrRP31** 5 mg/kg, IP once a day 5 days/week 6 weeks	Reduced food intake Reduced body weight Improved OGTT Leptin ↓	[183]
**DIO mice ♂** HFD: from 6th week, 18 weeks	**18-S4** (analog of PrRP31, agonist of GPR10 receptor) 0.5 mg/kg, SC 12 days	Reduced body weight	[185]
**C57BL/6 J mice ♂** HFD: 5th week, 47 weeks	**GUB03385** 1.250 nmol/kg, SC 7 days + 7 days wash-out	Reduced food intake Reduced body weight	[187]
	**NPFF**		
**C57BL/6 mice ♂** HFD: from 8th week, 12 weeks	**NPFF** 4 nmol/kg, IP 18 days	No decreased body weight No decreased food intake No decreased weight of AT glucose tolerance, AT insulin sensitivity ↑ mRNA *Npffr2* ↑	[216]
**C57BL/6N mice ♂** HFD: from 8th week, 4 months	**oct-1DMe** 10 mg/kg, SC 28 days	No decreased body weight No decreased food intake	[217]
	**TRH**		
**CD1 mice ♀** NS	**Synthetic TRH (5-Oxo-L-prolyl-L-histidyl-L-prolinamide monotartrate monohydrate)** 0.3 mg/kg, IP 26 days + 30 days wash-out	Reduced food intake Reduced body weight, however, increased body weight during wash-out period Leptin, cholesterol, TAG ↓	[262]

Abbreviations:* Acaca,* acetyl-CoA carboxylase; AT, adipose tissue; BAT, brown adipose tissue; CART, cocaine- and amphetamine-regulated transcript; DIO, diet-induced obesity; ERK, extracellular signal-regulated kinase; *Fasn,* fatty acid synthase; FFA, free fatty acids; HFD, high-fat diet; ICV, intracerebroventricular; ing, inguinal; IP, intraperitoneal; MCR, melanocortin receptor; MSH, melanocyte-stimulating hormone; myr, myristoyl; NS, not specified; *Npffr2,* neuropeptide FF receptor 2; oct, octanoyl; OGTT, oral glucose tolerance test; palm, palmitoyl; *Pgc-1α,* peroxisome proliferator-activated receptor-γ coactivator; PI3K, phosphoinositide 3-kinases; PrRP, prolactin-releasing peptide; SC, subcutaneous; TAG, triacylglycerols; TRH, thyrotropin-releasing hormone; Ucp1, uncoupling protein 1; WAT, white adipose tissue; ♂, male; ♀, female

**Table 2:** Pharmacological interventions of neuropeptides in rodent models of AD-like pathology

Animal model Starting age	Intervention Compound/dose/ injection/duration	Effect of memory/Behavioral test	Effect in hippocampus/cortex	Marker Increased ↑ Decreased ↓	Reference
	**Melanocortins**				
**TgCRND8 ♂, ♀** 20 weeks old	**α-MSH** 0.5 mg/kg IP daily 28 days	OF: normal anxiety, no effect on locomotion Y maze: preserved spatial memory	Increased synaptic plasticity	GAD67 ↑	[100]
**Sprague-Dawley rats ♂** Transient global cerebral ischemia NS age	**α-MSH** 0.5 mg/kg IP 30 min post-ischemia, and at 24, 48, 72, 96 h		Viable neurons in the CA1 pyramidal cell layer Higher number of viable neurons	GFAP-labeled cells ↓ intensity	[101]
**Mongolian gerbils ♂** Transient global brain ischemia NS age	**NDP-α-MSH** 340 µg/kg IP 2 × daily 11 days	MWM: improved learning and memory	Prevents DNA fragmentation in the hippocampal cells	BrdU-labeled cells ↑ BrdU-NeuN+ ↑ Zif268 ↑	[102]
**3xTg mice ♂** 12 weeks old	**NDP-α-MSH** 340 µg/kg IP daily 18 weeks +/- inhibitor HS024	MWM: improved learning and memory	Decreased Aβ pathology Reduced Tau phosphorylation Decreased apoptosis Reduced neuroinflammation	Aβ plaques ↓, p-APP Thr668 ↓ p-Tau Thr181, p-Tau Ser396, p-Tau Ser202 ↓ p-p38 ↓, Caspase-3 ↓ IL-1β↓, TNF-α ↓	[103]
**5XFAD mice** NS sex 5 and 7 months old **3xTg mice ♂** 9 and 12 months old	**NDP-α-MSH** 340 mg/kg IP daily 50 days	MWM: improved spatial memory	Microglial reactivity Reduced AD-related markers	Iba1 ↓ in 3xTg mice p-Tau Ser396, p-Tau Ser202, p38 MAPK ↓ in 5XFAD p-Tau Ser202, p38 MAPK ↓ in 3xTg	[104]
**Tg2576 ♂** 24 weeks old	**NDP-α-MSH** 340 µg/kg IP daily 50 days	MWM: improved spatial learning and memory	Decreased Aβ pathology Increased synaptic plasticity	Aβ plaques ↓ Zif268 ↑	[105]
**Tg2576 mice ♂** 24 weeks old	**NDP-α-MSH** 340 µg/kg IP daily 50 days	MWM: improves learning and memory	Decreased Aβ pathology Increased neurogenesis Reduced neuroinflammation	Aβ plaques ↓ BrdU+ ↑, Zif268 ↑, NeuN ↑ GFAP ↓	[106]
**APP/PS1 mice ♂** 6–7 months old	**D-Tyr MTII** ICV Alzet minipumps 2.4 nmol/day 28 days		Decreased Aβ pathology Reduced neuroinflammation Increased synaptic plasticity	Aβ plaques ↓ IL-1β ↓, Icam1 ↓ *Gfap* ↓, *Aif1* ↓ C3^+^ GFAP^+^ astrocytes ↓	[108]
	**CART peptide**				
**Sprague-Dawley rats ♂** NS age	**CARTp [54–102]** 25-100ng/rat/day 4 days CART antibody ICV daily	MWM: improved spatial memory	Increased CART-immunoreactive fibers in the hippocampus	CART-ir fibers ↑	[143]
**APP/PS1 mice ♂** 8 months old	**CART peptide** IV 0.5 µg/kg 10 days + IP 0.5 µg/kg 20 days	MWM: improved spatial memory	Decreased Aβ pathology Increased synaptic plasticity Reduced reactive oxygen species	Aβ plaques ↓ LTP ↑, SYP ↑ ROS ↓	[144]
**APP/PS1 mice ♂** 6 months old	**CART peptide** IV 0.5 µg/kg 10 days + IP 0.5 µg/kg 20 days	MWM: improved spatial memory	Decreased Aβ pathology Increased insulin signaling	Soluble Aβ ↓, Aβ enzymes ↓ Akt ↑	[145]
**APP/PS1 mice ♂** 8 months old	**CART peptide** IV 0.5 µg/kg 10 days + IP 0.5 µg/kg 20 days	MWM: improved spatial memory	Decreased Aβ pathology Activation of Aβ-degrading enzymes Decreased reactive oxygen species	Aβ plaques ↓ NEP, IDE ↑ ROS level ↓	[146]
**Sprague-Dawley rats ♂** Intrahippocampally injected with Aβ_1-42_ NS age	**CART** [55–102] Intrahippocampal injection 0.02 µg/hemisphere 5 days	MWM: improved spatial memory OF: improved locomotor activity	Decreased Aβ pathology Attenuated oxidative stress Decreased neuronal apoptosis	Aβ plaques, BACE1 ↓ MDA ↓ T-SOD, GSH, ATP, Nrf2, HO-1, NQO1 ↑ Bcl-2 ↑ Bax, caspase 3, caspase 9 ↑	[147]
	**PACAP**				
**APP(V717I) mice ♂** 3 months old	**PACAP38** IN 10 µg/day 5 days/week 3 months	NOR: improved memory	Increased nonamyloidogenic pathway of APP Increased expression of BDNF	Neuroprotective sAPPα ↑ soluble Aβ ↓ BDNF ↑	[158]
	**PrRP**				
**MSG mice ♂** 6 months old	**Palm-PrRP31** SC 5 mg/kg Liraglutide SC 0.2 mg/kg Daily, 14 days		Reduced Tau phosphorylation Increased insulin signaling	p-GSK3β(Ser9) ↑ p-Tau Ser396 ↓, p-Tau Thr231 ↓, p-Tau Thr212 ↓ p-PDK1(Ser241) ↑ p-Akt (Thr308), (Ser473) ↑	[188]
**Thy-Tau22 mice ♀** 7 months old	**Palm^11^-PrRP31** Alzet minipumps SC 5 mg/kg/day 2 months	Y maze: improved short-term working memory	Reduced Tau phosphorylation Increased synaptic plasticity Increased insulin signaling	p-GSK3β(Ser9) ↑, PP2A subC ↑ p- Tau Ser396 ↓, p-Tau Ser404 ↓, p-Tau Thr231 ↓ PSD-95 ↑, SYP ↑ p-Akt (Ser473) ↑, p-Akt (Thr308) ↑	[189] [54]
**APP/PS1 mice ♂** 7 months old **APP/PS1 mice ♂** 7 months old	**Palm^11^-PrRP31** SC 5 mg/kg Liraglutide SC 0.2 mg/kg Daily, 2 months **Palm^11^-PrRP31** SC 5 mg/kg Daily, 2 months		Decreased Aβ pathology Decreased neuroinflammation Reduced Tau phosphorylation Mild increase in neurogenesis Increased synaptogenesis Reduced Aβ in cerebellum Reduced microgliosis in cerebellum Decreased pro-inflammatory proteins in hippocampi Increased synaptogenesis Decreased apoptosis	Aβ plaques ↓ Iba-1 ↓, GFAP ↓ p-Thr231 ↓ DCX ↑ SYP ↑ Aβ plaques ↓ Iba-1 ↓ CD68 ↓, IFNγ ↓ Syntaxin 1A↑, SYP ↑, PSD95 ↑ Bax/Bcl2 ↓	[190]
	**NPFF**				
**Sprague-Dawley rats ♂** NS age	**NPFF** VTA 1, 2.5, 5, 7.5 or 10 µg/mice	Cages: reduced locomotion			[225]
**Wild-type mice** (NS strain) **♂** 3–4 months old	**NPFF** ICV 1.0 µg/mice ICV 10 µg/mice	MWM: spatial acquisition improved reduced			[226]
**C57BL/6J mice ♂** 3 months old	**1DMe** ICV 1 or 10 nmol	OL: impaired short-term memory MWM: impaired long-term memory			[227]
**CFLP mice ♂** NS age	**NPAF** ICV 1.0 µg/mice	Improved passive avoidance learning			[228]
	**CRH**				
**APP/PS1 mice ♂, ♀** 1 month old	**R121919 (CRH 1 receptor antagonist)** SC 20 mg/kg 150 days	MWM: improved spatial memory	Decreased Aβ plaque load Decreased activity of BACE Increased synaptogenesis	Aβ plaques ↓ BACE ↓ SYP ↑, MAP2 ↑	[244]
**Tg2576 mice chronically stressed ♂, ♀** 4 months	**Antalarmin (CRH 1 receptor antagonist)** 20 mg/kg in drinking water, 6 months	EPM: decreased anxiety-like behavior Y -maze: improved working memory	Decreased level of Aβ in chronically stressed mice	Aβ plaques ↓, Aβ_42_ ↓	[245]
**PS19 mice ♂, stressed for 1 month during the treatment** 7 months	**NBI 27914 (CRH 1 receptor antagonist)** SC 10 mg/kg 6 day/week 4 weeks	Fear conditioning: improved impairment in fear-associated memory	Attenuated Tau hyperphosphorylation Prevented neuronal loss	AT8 ↓, PHF1 ↓ NeuN ↑	[246]
**Sprague-Dawley rats ♂, ♀ exposed to Isolation-restraint stress** 18 months	**R121919 or antalarmin** 20 mg/kg mixed in chow diet 3 months	OF: decreased anxiety NOR: improved memory MWM: improved spatial memory	Increased spine density in cortex Increased synaptic density	Spine density ↑ Synaptic density ↑	[247]

Abbreviations: 1DMe, stable analog of neuropeptide; Aβ, amyloid β; *Aif*, allograft inflammatory factor 1; APP, amyloid precursor protein; AT8, antibody pTau Ser202&Thr205; BACE1, β-site APP cleaving enzyme 1; Bax, proapoptotic protein; Bcl-2, antiapoptotic protein; BDNF, brain-derived neurotrophic factor; BrdU+, bromouridine positive neurons; CART, cocaine- and amphetamine-regulated transcript; CD68, a scavenger receptor extensively increased in highly reactive microglia; CDK-5, cyclin dependent kinase 5; CRH, corticotropin-releasing hormone; DCX, doublecortin; EPM, elevated plus maze; FF, 3xTg triple transgenic mice; GAD67, glutamic acid decarboxylase 67; GFAP, glial fibrillary acidic protein; GSH, glutathione; GSK-3β, glycogen synthase kinase 3β (pSer9: inhibition, pTyr216: activation); HFD, high-fat diet; HO-1, heme oxygenase-1; Iba1, ionized calcium-binding adaptor molecule 1; Icam1, intercellular adhesion molecule 1; ICV, intracerebroventricular; IDE, insulin-degrading enzyme; IFNγ, interferon γ; IL, interleukin; IN, intranasal; IP, intraperitoneal; IV, intravenous; LTP, long-term potentiation; MAP2, microtubule-associated protein 2; MAPK, mitogen-activated protein kinase; MDA, malondialdehyde; MSG, monosodium glutamate; MSH, melanocyte-stimulating hormone; MWM, Morris water maze; NEP, neprilysin; NeuN neuronal nucleus, marker of mature neurons; NOR, novel object recognition test; NPAF, neuropeptide AF; NPFF, neuropeptide FF; NQO1, NADPH quinone oxidoreductase 1; Nrf2, Nuclear erythroid-2-related factor 2; NS, not specified; OF, open field; OL, object location; PACAP, pituitary adenylate cyclase-activating peptide; PDK1, phosphoinositide-dependent kinase 1; PHF1, antibody pTau Ser396&Ser404; PS1, presenilin 1; PP2A, subC protein phosphatase 2A subunit C; palm, palmitoylated; PrRP, prolactin-releasing peptide; PSD95, postsynaptic density protein 95; ROS, reactive oxygen species; SC, subcutaneous; SYP, synaptophysin; T-SOD, superoxide dismutase; VTA, ventral tegmental area; Zif268, immediate early response gene; ♂, male; ♀, female.

### Cocaine- and amphetamine-regulated transcript peptide (CARTp)

In 1995, Douglass et al. [109] discovered that acute administration of cocaine and amphetamine increased the expression of specific mRNAs; thus, they designated this mRNA CART. The structure of CARTp was identified later, in 1998, when Thim et al. [110] described two CARTp isoforms isolated from the rat hypothalamus, CARTp [55–102] and CARTp [61–102]; both were subsequently confirmed to be biologically active peptides [111–113]. The newly described CARTp was linked to a previously isolated peptide from the ovine hypothalamus with unknown functions [114]. CARTp is evolutionarily conserved across species; there is 95% amino acid identity between active rat and human CART peptides [109,115,116]. The CARTp receptor has not yet been identified. However, there is evidence suggesting that the CARTp receptor could be a GPCR since CARTp [55–102] can inhibit voltage-dependent Ca^2+^ channels in primary hippocampal neurons [117]. This inhibitory effect was blocked in cells treated with pertussis toxin, suggesting that CARTp [55–102] mediates this inhibition through the activation of the G proteins G_i/o_. CARTp activated extracellular signal-regulated kinase (ERK) in the mouse pituitary tumor cell line AtT20, where specific binding was observed [118,119]. Our group reported the specific binding of CARTp [61–102] in the nanomolar range to rat pheochromocytoma PC12 cells [120]. The number of binding sites is increased fivefold in PC12 cells differentiated to a neuronal phenotype with nerve growth factor (NGF). Our subsequent study demonstrated the activation of the stress-activated protein kinase/c-jun NH_2_-terminal kinase (SAPK/JNK) pathway in response to CARTp stimulation in PC12 cells [121]. In 2020, Yosten et al. [122] identified the orphan receptor GPR160 as a potential receptor for CARTp, although specific binding of CARTp to GPR160 was not demonstrated in these studies. Our next study did not confirm the presence of GPR160 in PC12 cells [123]. Moreover, no specific binding of CARTp to THP1 cells with high endogenous GPR160 expression or cells transfected with GPR160 was detected. While GPR160 might play a role in CARTp signaling, further studies are needed to identify the receptor for CARTp.

CART is among the most predominant transcripts in the hypothalamus [124], with both CART mRNA and CART immunoreactivity observed in distinct nuclei across the brain, especially in the hypothalamus in the ARC, LHA and PVN, nucleus accumbens, or pituitary, as well as in the periphery, e.g., adrenal glands [125], islets of Langerhans [126] and gut [127]. According to the distribution of CARTp in various brain regions implicated in food intake regulation, it has been proposed that CARTp play a role in the control of eating behavior [125,128]. Since CARTp-deficient mice exhibit late-onset obesity and impaired insulin secretion, these findings were confirmed [129,130]. Subsequent studies of ICV-administered CARTp fragments in rats or mice showed potent food intake-lowering effects, accompanied by the inhibition of NPY neurons [111,131,132]. Moreover, Kristensen et al. demonstrated the importance of leptin in the activation of CART mRNA expression in the ARC. This was supported by the findings of another study in which animals with disrupted leptin signaling were used, which showed almost no expression of the peptide in the brain [133]. The increase in the expression of CARTp after leptin administration [134], the presence of leptin receptors on CART-positive neurons in various regions of the hypothalamus [135], the colocalization of CART with anorexigenic α-MSH in neurons of the ARC [136] and the concomitant modulatory effect of the release of the energy homeostasis regulator TRH from the pituitary [137,138] indicate the effects of CARTp on feeding and energy expenditure. CARTp is also implicated in CCK-induced satiety [139]; moreover, coadministration of CART peptide (ICV) and CCK (IP) synergistically reduced food intake in fasted mice [140]. Six-day-long infusion of CARTp [55–102] into the right lateral cerebral ventricle of DIO rats resulted in a decrease in food intake and body weight loss [141]. In addition to the reduced food intake, the ICV injection of DIO rats with CARTp [55–102] showed enhanced lipid metabolism, as indicated by increased plasma levels of nonesterified fatty acids, suggesting the hydrolysis of stored triglycerides [142].

The first indications of the potential neuroprotective effects of CARTp were described in 2011, when ICV injection of CARTp for four consecutive days in rats resulted in significantly improved spatial learning and memory in the MWM test [143]. Furthermore, immunohistochemical data have shown significantly increased CART-immunoreactivity in brain areas involved in learning and memory in rats after four days of training in the MWM test [143]. The neuroprotective effects of CARTp on the pathology of AD were studied by Xu et al. They observed Aβ plaque-associated CART immunoreactivity in the hippocampus and cortex of 8-month-old APP/PS1 mice as well as in the cortex of human AD patients [144]. Chronic CARTp treatment attenuated memory deficits in APP/PS1 mice and improved synaptic ultrastructure and long-term potentiation of neurons. Additionally, it reduced reactive oxygen species but did not have an impact on the reduction in Aβ [144]. A subsequent study showed significantly decreased levels of soluble Aβ_1-40_ and Aβ_1-42_ in the hippocampus of APP/PS1 mice after CARTp treatment [145]. The number of Aβ plaques was reduced due to activation of Aβ-degrading enzymes such as neprilysin, insulin-degrading enzyme, and low-density lipoprotein receptor-related protein 1 [146]. Moreover, CARTp reduced levels of reactive oxygen species in the hippocampus of APP/PS1 [146]. Similar results were observed in rats injected intrahippocampally with Aβ_1-42_; Aβ induced a reduction in CART-immunoreactive fibers, but this was prevented by 5-day pretreatment with CARTp injected to the hippocampus [147]. CARTp further improved spatial memory in MWM, decreased oxidative stress and attenuated neuronal apoptosis [147].

Taken together, these findings suggest that CARTp could be used as an antiobesity or neuroprotective drug in the treatment of neurodegenerative diseases. Its beneficial effects on mouse models of obesity and AD-like pathology are summarized in Tables 1 and 2. However, understanding the exact mechanism of action of CARTp in models of obesity and neurodegeneration is necessary, and discovering of the CARTp receptor is essential for this purpose.

### Pituitary adenylate cyclase-activating peptide (PACAP)

PACAP is a 38-amino-acid or 27-amino-acid neuropeptide produced and released both in the periphery and the CNS, especially in the VMN, or in the LHA and PVN in the hypothalamus [148–150]. PACAP binds to three GPCRs: PAC1R, VPAC1, and VPAC2 [151]. Its metabolic pathways are linked to the regulation of body weight and the development of obesity and metabolic syndrome. In addition to appetite regulation, PACAP also increased thermogenesis in mice [22,23]. The importance of PACAP is stressed by its involvement in the leptin-induced decrease in food intake and increased thermogenesis as a marker of increased energy expenditure [152]. Moreover, PACAP stimulates POMC neurons in the ARC but inhibits NPY/AgRP [153]. A study of PACAP-null mice revealed decreased survival of newborn mice when the mice were housed at room temperature. Moreover, these mice had decreased body weight compared to that of their wild-type littermates due to a decreased amount of WAT [154]. Even though PACAP is involved in the regulation of energy homeostasis and could be considered a possible target for obesity treatment, the direct antiobesity effects of PACAP have still not been well explored [155].

High expression of PACAP was detected in the hippocampus, particularly in the dentate gyrus (DG), where increased synaptic transmission was observed after PACAP treatment [156]. This finding highlights the possible neuroprotective effects of PACAP in the treatment of dementia. Postmortem analysis of brains from patients with AD revealed a negative correlation between the level of Aβ plaques and PACAP, and between the level of PACAP and tau pathology (according to Braak stages of AD severity) [157]. As summarized in Table 2, 3-month-long intranasal application of PACAP to APP(V717I) mice harboring the London mutation improved short-term memory in the novel object recognition test, decreased the level of soluble Aβ, and increased the levels of neurotrophin BDNF [158].

### Prolactin-releasing peptide (PrRP)

PrRP is a hypothalamic neuropeptide with a misleading name [159]. Shortly after its discovery, the initially described stimulation of prolactin was questioned; nevertheless, the name remained [160,161]. In organism, two equally active isoforms can be found: PrRP31 with 31 amino acids, or its shorter analog PrRP20 with an identical C-terminal sequence. The last two amino acids at the C-terminus, Arg-Phe-amide, are important for preserving the binding affinity of PrRP to its receptor and proper biological activity [159,162–164]. PrRP was identified as a ligand of GPR10 (also known as hGR3 or UHR1) [159]. It displayed high affinity for the receptor type 2 for neuropeptide FF (NPFFR2), which is another neuropeptide from the RF-amide family [165]. Studies investigating the distribution of PrRP or its receptor in the organism revealed high expression of PrRP in centers implicated in food intake regulation, such as the hypothalamus (DMN, VMH, or PVN) or in the NTS in the brainstem [24,166–169]. Subsequent studies confirmed that ICV injection of PrRP significantly reduced food intake in free-fed rats and decreased body weight [24,164,170]. The weight loss observed was greater than that corresponding to a reduction in food intake alone, suggesting that increased energy expenditure also contributes to the weight loss induced by PrRP administration [171]. The importance of PrRP in food intake regulation and energy balance is emphasized by the fact that PrRP expression is directly stimulated by leptin, the main regulator of energy homeostasis [172]. PrRP also mediates the anorexigenic effect of peripheral anorexigenic CCK [173]. Moreover, PrRP-deficient [174] or GPR10-deficient [175,176] mice develop late-onset obesity and decreased energy expenditure.

The peripheral injection of natural PrRP31, a convenient route for potential anti-obesity treatment, does not decrease food intake in fasted mice [177]. Thus, a series of PrRP20 or PrRP31 lipidized with fatty acids of different lengths (from octanoyl to stearoyl [stear]) at the N-terminus were designed. Lipidization did not influence binding to the GPR10 receptor; moreover, these analogs exhibited increased affinity for NPFFR2 [177]. Only myr-PrRP20, myr-PrRP31, palm-PrRP31 and stear-PrRP31 significantly reduced food intake in overnight fasted mice after acute subcutaneous (SC) application [177]. In free-fed rats, palm-PrRP31 administered for three consecutive days significantly reduced food intake after SC or intraperitoneal (IP) injection at a dose of 5 mg/kg. A comparable effect was observed after intravenous (IV) administration even at a dose of 0.1 mg/kg [178]. Finally, myr-PrRP20 or palm-PrRP31 was chronically SC injected into DIO mice for 2 weeks; this treatment resulted in significant weight loss and improved metabolic parameters related to obesity, such as decreased leptin or insulin levels [177]. A comprehensive study of different lipidized analogs of PrRP31 performed by Pražienková et al. [179] defined an analog of PrRP31 palmitoylated at position 11 (where arginine is substituted with lysine) through a γ-glutamic acid linker (palm^11^-PrRP31) as an analog with improved bioavailability. Two-week-long treatment of DIO mice significantly reduced body weight, improved metabolic parameters, and decreased *de novo* lipogenesis. Both analogs also increased the expression of uncoupling protein 1 in brown adipose tissue (BAT), suggesting increased energy expenditure in these mice [177,179]. A subsequent study of chronic SC administration of palm^11^-PrRP31 investigated the possible yo-yo effect after termination of the treatment [180]. One group of DIO mice was SC injected for 28 days with palm^11^-PrRP, while the second group received palm^11^-PrRP31 for 14 days and then saline for the subsequent 14 days. As expected, 28 days of treatment significantly reduced the body weight of the mice. A comparable body-weight reduction was observed in the palm^11^-PrRP31-saline group after 2 weeks of treatment. Interestingly, there was no body weight gain within the subsequent 2 weeks of saline administration [180]. Similar trends toward a reduction in food intake, decreased body weight, and improvements in metabolic parameters, such as decreased levels of glucose, leptin, or insulin, were observed in several rat DIO models [181–183]. The effects of palmitoylated-PrRP31 analogs in different models of obesity were reviewed by Mráziková et al. [184]. Another series of modified PrRP analogs with multiple ethylene glycol-fatty acid (MEG-FA) stapling platform was described by Pflimlin et al. [185]. The lead compound 18-S4, a selective agonist of GPR10, showed improved bioavailability and stability in serum. Twelve-day-long SC treatment of DIO mice resulted in a significant decrease in body weight [185]. On the other hand, several studies have described the importance of dual agonism toward GPR10 and NPFFR2 for the full antiobesity effect of lipidized PrRP31 analogs [177,179,186,187]. Alexopoulou et al. designed different series of lipidized PrRP analogs with selectivity for either GPR10 or both GPR10 and NPFFR2. Only analogs with dual agonist effect had potent antiobesity effects. Moreover, body weight did not increase after termination of treatment [187].

Palm-PrRP31 also significantly decreased the body weight of mice with obesity induced by monosodium glutamate (MSG), which is repeatedly SC injected to newborn mice [188]. In addition to obesity, MSG-obese mice with pre-diabetes, leptin and insulin resistance exhibited central insulin resistance leading to increased hippocampal Tau hyperphosphorylation. This effect was attenuated by palm-PrRP31 treatment through the activation of the central insulin signaling cascade and inhibition of glycogen-synthase kinase 3β (GSK-3β), the main kinase of the Tau protein [188]. The effect of palm^11^-PrRP31 on the attenuation of Tau hyperphosphorylation was further observed in Thy-Tau22 mice overexpressing mutated human Tau with accelerated hyperphosphorylation [189]. One month of SC infusion of palm^11^-PrRP31 attenuated Tau hyperphosphorylation at different epitopes in the hippocampus, increased the expression of markers of synaptic plasticity, and improved spatial working memory in the Y-maze test [189]. Potential neuroprotective effects of palm^11^-PrRP31 were also demonstrated in APP/PS1 mice, in which 2 months of treatment significantly decreased the number of Aβ plaques in the hippocampus, cortex [54], and cerebellum [190]. Tau phosphorylation at different epitopes was also attenuated after palm^11^-PrRP31 treatment in the hippocampus [54]. Aβ plaques colocalize with microgliosis and astrocytosis, whose levels also decrease after treatment with palm^11^-PrRP31 [54,190]. Further analysis revealed changes in the distribution of various lipids, mainly gangliosides (GM2 36:1, GM3 36:1) and phosphatidylinositols (PI 38:4, 36:4), around Aβ plaques; moreover, the lipid profile normalized after treatment with palm^11^-PrRP31 [191]. Moreover, palm^11^-PrRP31 increased synaptic plasticity [54,190], neurogenesis manifested as an increase in doublecortin-positive cells in the hippocampal DG [54], and decreased apoptosis in the hippocampus [190]. Recently, a beneficial effect on adult neurogenesis, which was impaired in DIO mice, was described [192]. The possible implication of PrRP and GPR10 for proper brain function was stressed by the recent finding that decreased GPR10 receptor levels were observed in patients with AD [193].

Studies in mouse models of obesity (summarized in Table 1) or mouse models of amyloidosis or tauopathy (summarized in Table 2) have shown promising potential antiobesity and neuroprotective properties of palmitoylated PrRP31 analogs.

### Neuropeptide FF (NPFF)

The octapeptide NPFF and the octadecapeptide neuropeptide AF (NPAF) were first isolated from bovine brain tissue in 1985 [194]. In rodents, NPFF was found to be highly expressed in the brainstem in the NTS, dorsal horn of the spinal cord, and hypothalamus between the DMN and VMN, with neuronal projections to the PVN [195–198]. Autoradiographic studies have shown that NPFF receptors are present in memory-related brain regions, such as the amygdala, hippocampus, bed nucleus of the stria terminalis, and cortical regions [199,200].

The function of NPFF is associated with two types of GPCRs–NPFFR1 and the NPFFR2 receptor [200]. The presence of the NPFFR1 and NPFFR2 mRNAs in the medulla, lateral hypothalamus, and thalamus indicates that NPFF participates in the regulation of responses to painful stimuli [200,201]. ICV administration of NPFF to mice reduced the analgesic effects of morphine and lowered the pain threshold, indicating that NPFF likely has anti-opioid effects. This is further supported by the fact that ICV administration of the anti-NPFF antiserum increased opiate-induced analgesia and restored sensitivity to morphine in mice that had already developed tolerance to its analgesic effects [202]. NPFF was found to be physiologically more active than NPAF in decreasing tail-flick latency in rats and it also attenuated the prolongation of tail-flick latency induced by morphine [194].

Multiple NPFF agonists and antagonists resistant to peptidases, which cause rapid inactivation of NPFF, were synthesized and tested. The analog 1DMe ([D-Tyr^1^, (N-Me)-Phe^4^] NPFF) has an affinity comparable to that of NPFF [203] and has been shown to inhibit morphine-induced analgesia in mice [204]. Several candidates for NPFF receptor agonists and antagonists have been synthesized, but no antagonist has demonstrated high selectivity and activity [205]. Centrally administered RF9, a reported NPFF receptors antagonist, was found to block hyperalgesia after prolonged administration of opioids [206] and antagonize the hypothermic effects induced by the selective agonists of NPFFR1 and NPFFR2 in mice [207]. However, RF9 did not reverse the anorectic effect of the agonist [Tyr^1^]NPFF, and its biological effects appear to be more agonistic than antagonistic [208].

Since many opioid agonists have been shown to increase food intake, NPFF was also investigated in this context. ICV administration of NPFF rapidly reduced food intake in dose-dependent manner in fasted rats [209,210]. This anorexigenic effect was initially attributed to increased water intake [210]; however, in other studies, this dipsogenic effect was no longer reported [211]. The regulation of feeding is likely through modulation of hypothalamic neurons [212]. Furthermore, both the central administration of NPFF [213] or NPAF [214] reduced food intake in fasted chicks. The centrally injected agonist [Tyr^1^]NPFF significantly lowered food intake in fasted mice [215]. Interestingly, ICV administration of NPFF caused a significant decrease in food intake in both wild-type mice and mice lacking GPR10 receptor. The ability of NPFF to reduce food intake in the GPR10 KO mice suggests that NPFFR2 expression is maintained in these animals [173]. NPFF was reported to promote the activation of adipose tissue macrophages (ATMs), which have an impact on the development of obesity-induced metabolic diseases, to an alternative M2 activation state, which is metabolically beneficial and is activated in lean adipose tissue. In ATMs, NPFFR2 is expressed in both humans and mice. Plasma levels of NPFF are decreased in obese patients and mice on a HFD and restored after caloric restriction. In this study, HFD-fed mice treated IP with NPFF did not decrease body weight, food intake, or the weight of adipose tissue [216]. In our recent study, involving lipidized NPFF and NPAF analogs, we observed only a slight anorexigenic effect on fasted lean mice following the SC administration of octanoylated-1DMe analog. In mice fed a HFD, long-term treatment did not result in reduced food intake or body weight [217]. Alongside its anorexigenic effect, NPFF was implicated in blood pressure regulation [218,219] as well as body temperature [220,221].

Multiple experiments suggest a role for NPFF in cognitive functions due to its opioid-modulating properties, as the endogenous opioid system is involved in the modulation of behavior. A single ICV injection of NPFF reduced the expression of morphine-induced sensitization in rats with a conditioned place preference [222], as did the rewarding effects of cocaine or amphetamine [223,224]. NPFF also inhibited hyperlocomotor activity of cocaine-induced sensitization in mice [223]. RF9 reversed the inhibitory effect of NPFF but did not affect amphetamine- or saline-conditioned rats [224].

Injection of NPFF in the ventral tegmental area reduced the increase in locomotor activity induced by novelty exposure [225]. Moreover, NPFF impairs spatial acquisition by significantly reducing spatial learning in the MWM [226]. 1DMe, injected via the ICV, induced delayed hyperlocomotion and mildly impaired both short-term and long-term spatial memory without affecting contextual fear memory in mice [227]. Additionally, the NPAF has a stimulatory effect on memory consolidation in passive avoidance learning [228]. Serum NPFF levels are significantly elevated in patients with spinal cord injury [229], which is associated with cognitive impairment [230]. These data suggest that the NPFF may have prognostic value for predicting cognitive impairment in patients with spinal cord injury, as its peripheral levels are normally limited and could arise due to leakage from CNS tissues [231].

Taken together, these results demonstrate the small but complex influence of the NPFF system on mouse behavior and cognitive functions, as summarized in Table 2. However, only a limited number of studies have been conducted in this area, and further experiments need to be explored.

### Corticotropin-releasing hormone (CRH)

The hypothalamic CRH is an important physiological activator of POMC-derived hormones, such as ACTH and β-endorphins [232–234]. CRH consists of 41 amino acids, has an amidated C-terminus, is widely distributed in the CNS [235], for example, in the hypothalamus in the PVN with projections to the ME, in the cortex or in the hippocampus. CRH signals through two receptors, CRH-R1 and CRH-R2, both belonging to the GPCR family and widely distributed in both the CNS and the periphery [236].

CRH is considered an anorexigenic compound. However, its direct involvement in obesity treatment in preclinical models has not yet been proven. When administered via the ICV, CRH significantly decreased food intake in rats [237] and rhesus monkeys [238], after acute administration. Seven-day-long infusion of CRH resulted in significantly reduced food intake in rats followed by decreased body weight and increased thermogenesis in BAT [237].

Early dysregulation of the hypothalamic–pituitary–adrenal (HPA) axis or stress axis was observed in patients with sporadic AD, followed by increased secretion of glucocorticoids. Hormones of the HPA axis and their receptors are proposed to be involved in AD; therefore, they could be targets for treating neurodegenerative diseases (reviewed previously [239]). Intrahippocampal CRH administration to mice exposed to acute stress via the food shock test or via an environment with predator odor increased hippocampal long-term potentiation and strengthened synaptic plasticity. Thus, CRH could be implicated in stress-enhanced memory consolidation during stress conditions [240]. In patients with AD, a reduced level of CRH was observed [241], whereas the number of CRH receptors increased [242]. In AD brain tissues, reduced concentrations of CRF-like immunoreactivity are accompanied by a significant reciprocal increase in CRF receptor binding within affected cortical areas. This increase in CRH binding correlates significantly with the decreased levels of CRH [243].

Further research on this phenomenon revealed that in different mouse models of AD, for example, in APP/PS1 mice [244], Tg2576 mice [245], and PS19 mice, a model of tauopathy [246], antagonists of CRH receptor 1 (CRH-R1) improved memory deficits and decreased Aβ and Tau hyperphosphorylation. Moreover, a CRH-R1 antagonist also prevents memory deficits and synaptic loss in 18-month-old rats with stress-induced memory deficits [247].

Hormones of the HPA axis and their receptors are proposed to be involved in AD; therefore, they could be targets for treating the neurodegenerative diseases reviewed previously [239], and are summarized in Table 2.

### Thyrotropin-releasing hormone (TRH)

The hypothalamic tripeptide TRH (pGlu-His-Pro-NH_2_) expressed in the PVN [248] is directly stimulated by leptin [249] or POMC-derived peptides [250], and inhibited by NPY/AgRP [251]. TRH is synthesized from a larger inactive precursor pro-TRH through a series of post-translational modifications [252]. This hormone exerts its effects through GPCR receptors, which are categorized as TRH-R1, TRH-R2, and TRH-R3. They exhibit species-specific variations; in humans, TRH-R1 is the unique type, while rodents express a second subtype, TRH-R2 and birds express TRH-R3 together with TRH-R1 [253].

TRH is known as the primary regulator of the hypothalamic-pituitary-thyroid axis, which is important for maintaining energy expenditure and body weight and is active even in states of leptin resistance associated with obesity [254]. Moreover, in a state of negative energy balance, the levels of TRH decrease [255,256]. Conversely, in DIO rats, the level of TRH was significantly increased [254]. Different routes of TRH application (IV, ICV, and SC) resulted in significantly reduced food intake and increased body temperature [25,257,258]. It has been described that TRH inhibits both food and water intake [259]. For example, the short-term reduction, with no concurrent reduction in body weight, occurs in rats following SC administration of TRH at dark onset [25]. Additionally, in another study, TRH decreased water intake when injected ICV [260]. However, in long-term treatment, with TRH administered twice daily for 5 days, it was observed that TRH did not reduce food intake, but instead, it increased water intake [25]. The reason for the stimulated water consumption is not clear. TRH administered both ICV and parenterally suppressed stress-induced eating. This effect was partially reversed by ICV administration of the long-acting synthetic enkephalin analog, suggesting that TRH and endogenous opiates may have a mutually antagonistic effect on ingestive behavior [261]. As shown in Table 1, chronic IP application of TRH to DIO mice reduced food intake and body weight, and improved metabolic parameters related to obesity, such as decreased levels of leptin, triglycerides, or cholesterol [262].

The role of TRH in AD is not well known; however, compared with healthy elderly controls, AD patients were shown to have decreased levels of TRH in the hippocampus [263]. A peptide analog of TRH, MK-771, improved spatial memory in a rat model of AD with medial septal lesions [264]. Moreover TRH could be implicated in the increased excitability of hippocampal CA1 neurons [265]. On the other hand, in a model of early-stage AD induced by intrahippocampal injection of okadaic acid, which enhances the activity of the Tau kinase GSK-3β and increases Tau phosphorylation, the level of TRH increases in the brain, as does the level of TRH in the blood serum [266].

## Conclusions and future directions

The use of anorexigenic neuropeptides for obesity or neurodegeneration treatment is still under investigation. Potential therapies targeting hypothalamic neuropeptide systems are in intensive preclinical research and have shown promising results for weight reduction, improvement of metabolic parameters, and amelioration of the loss of memory, neuroinflammation, or neurogenesis associated with neurodegenerative diseases.

**Figure 2:**
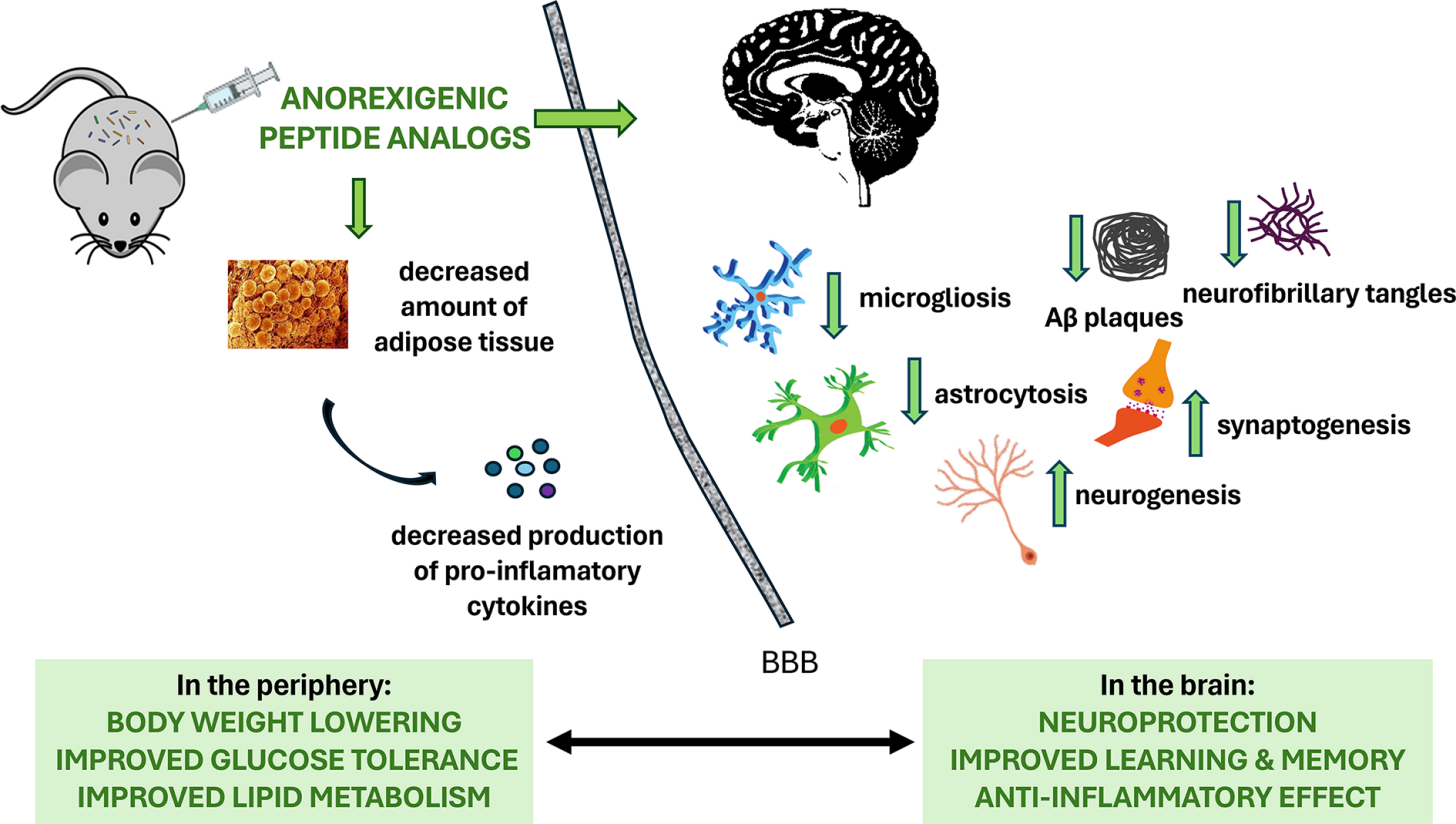
Scheme of beneficial effects of anorexigenic neuropeptides or their modified analogs in the treatment of obesity and neurodegeneration Administration of anorexigenic neuropeptides or their modified analogs reduces food intake, resulting in weight loss and decreased white adipose tissue, known for pro-inflammatory cytokine secretion. This treatment further mitigates neuroinflammation, characterized by microgliosis, and astrocytosis, lowers levels of amyloid-β (Aβ), and diminishes neurofibrillary tangles formed by hyperphosphorylated Tau protein in the brain. Additionally, it promotes synaptogenesis and neurogenesis, leading to improved learning and memory. BBB: blood brain barrier.

While the hypothalamic ARC is not entirely isolated from the peripheral circulation, targeting central neuropeptide systems in other CNS regions requires overcoming the blood–brain barrier. This often requires high drug doses, elevating the risk of possible side effects. Therefore, anorexigenic neuropeptides and their peptide-based analogs pose challenges when administered through injectable options. To achieve a central biological effect on their receptors after peripheral administration, it is necessary to modify neuropeptides to enhance their stability and bioavailability. In this review, we showed that anorexigenic neuropeptides, such as melanocortins, CARTp, PrRP, NPFF, PACAP, CRH and THR and their analogs not only decrease body weight, lower blood glucose levels, or ameliorate lipid profiles, but also improve cognitive impairment and the hallmarks of AD-like pathology in preclinical models (Figure 2).

Anorexigenic neuropeptides hold promise for addressing both obesity and neurodegeneration by modulating food intake, energy homeostasis, and displaying neuroprotective properties. Despite this potential, translating these discoveries into clinical practice presents significant challenges, including issues related to central nervous system access, side effects, and efficacy. These neuropeptides offer a promising avenue for developing neuropeptide receptor ligands with diverse pharmacological properties, and several analogs of anorexigenic peptides are currently undergoing preclinical trials. However, several obstacles persist, such as limited delivery to the brain and the need for comprehensive evaluation of their physiological effects, which may complicate their use in clinical trials. Nevertheless, they represent a valuable resource for developing novel pharmacological tools and therapeutic leads in both health and disease. However, targeting neuropeptides remains challenging due to the site of action and the route of administration.

## Abbreviations

AD: Alzheimer’s disease
AgRP: agouti-related peptide
ARC: arcuate nucleus
BAT: brown adipose tissue
CART: cocaine- and amphetamine-regulated transcript
CNS: central nervous system
ERK: extracellular signal-regulated kinase
GPAP: glial fibrillary acidic protein
HPA: hypothalamic–pituitary–adrenal
IL: interleukin
ME: media eminence
MEG-FA: multiple ethylene glycol-fatty acid
NPAF: neuropeptide AF
NPFF: neuropeptide FF
NPY: neuropeptide Y
PACAP: pituitary adenylate cyclase-activating peptide
POMC: pro-opiomelanocortin
PrRP: prolactin-releasing peptide
TRH: thyrotropin-releasing hormone
WAT: white adipose tissue
